# Higher dietary salt intake is associated with microalbuminuria, but not with retinopathy in individuals with type 1 diabetes: the EURODIAB Prospective Complications Study

**DOI:** 10.1007/s00125-014-3367-9

**Published:** 2014-08-30

**Authors:** Lian Engelen, Sabita S. Soedamah-Muthu, Johanna M. Geleijnse, Monika Toeller, Nish Chaturvedi, John H. Fuller, Casper G. Schalkwijk, Coen D. A. Stehouwer

**Affiliations:** 1 Department of Internal Medicine, Maastricht University Medical Centre, Universiteitssingel 50, 6200 MD Maastricht, the Netherlands; 2 CARIM School for Cardiovascular Diseases, Maastricht University Medical Centre, Maastricht, the Netherlands; 3 Top Institute Food and Nutrition, Wageningen, the Netherlands; 4 Division of Human Nutrition, Wageningen University, Wageningen, The Netherlands; 5 Heinrich-Heine-University Düsseldorf, Düsseldorf, Germany; 6 International Centre for Circulatory Health, National Heart and Lung Institute, Imperial College London, London, UK; 7 Department of Epidemiology and Public Health, Royal Free and University College London Medical School, London, UK

**Keywords:** Albuminuria, Dietary salt intake, Microvascular complications, Potassium excretion, Retinopathy, Sodium excretion, Type 1 diabetes

## Abstract

**Aims/hypothesis:**

High dietary salt intake has been associated with elevated BP and may also have a deleterious effect on microvascular complications. We studied the cross-sectional associations between dietary salt intake (estimated from 24 h urinary sodium excretion) and urinary potassium excretion on the one hand, and the prevalence of microvascular complications on the other, in individuals with type 1 diabetes.

**Methods:**

We measured sodium and potassium concentrations in two 24 h urine samples in 1,212 individuals with type 1 diabetes (40 ± 10 years old, 51% men) who participated in the EURODIAB Prospective Complications Study. We used multiple logistic regression analyses to investigate associations between dietary salt intake and microvascular complications adjusted for age and sex, and additionally for BMI, smoking, urinary potassium excretion, antihypertensive medication and physical activity, and total energy, protein, alcohol, saturated fat and fibre intake.

**Results:**

After full adjustment, 1 g/day higher dietary salt intake was positively associated with the presence of microalbuminuria (OR 1.06 [95% CI 1.01, 1.10]), but not macroalbuminuria (OR 0.99 [95% CI 0.94, 1.05]), non-proliferative retinopathy (OR 1.00 (95% CI 0.96, 1.04]) or proliferative retinopathy (OR 1.02 (95% CI 0.95, 1.08]). After excluding individuals with cardiovascular disease and/or antihypertensive medication (*n* = 418), we found a non-significant association with microalbuminuria (OR 1.04 [95% CI 0.99, 1.10]) and macroalbuminuria (OR 1.05 [95% CI 0.96, 1.16]). The association between dietary salt intake and microalbuminuria was stronger in individuals with a BMI above 25 kg/m^2^ (OR 1.11 [95% CI 1.04, 1.18]) than in those with BMI below 25 kg/m^2^ (OR 1.03 [95% CI 0.97, 1.09]). No significant associations were found between urinary potassium excretion and microvascular complications.

**Conclusions/interpretation:**

In individuals with type 1 diabetes, higher dietary salt intake, as determined by 24 h urinary sodium excretion, may be positively associated with microalbuminuria, particularly in overweight individuals.

**Electronic supplementary material:**

The online version of this article (doi:10.1007/s00125-014-3367-9) contains peer-reviewed but unedited supplementary material, which is available to authorised users.

## Introduction

Nutritional advice is important in preventing or slowing the rate of development of diabetes-related cardiovascular disease (CVD). Nutritional advice that favourably affects cardiovascular risk factors, such as BP, may also have a favourable effect on microvascular complications such as retinopathy and nephropathy [1, 2]. Lowering dietary salt intake is known to reduce BP [3].

Several large cohort studies conducted in the general population have investigated associations between dietary salt intake and prevalent microalbuminuria. In these studies dietary salt intake was estimated using either 24 h urinary sodium excretion [4–6], which is currently regarded as the reference method, or food frequency questionnaire (FFQ) [7, 8]. Both null [4, 8] and positive [5–7, 9] associations have been found between dietary salt intake and prevalent microalbuminuria in the general population.

In contrast, a smaller study on 270 individuals with type 2 diabetes in which a spot urine sample was used to estimate daily salt intake showed a ‘J-shaped’ association with albuminuria [10]. A J-shaped association was also found between 24 h urinary sodium excretion and all-cause mortality in individuals with type 1 diabetes [11]. In this Finnish cohort, low sodium excretion (the 10th percentile of the population) was also associated with the development of end-stage renal disease [11]. The association between dietary salt intake and (early) microvascular complications in individuals with type 1 diabetes, however, has not yet been established, except for one prospective study [12]. This study showed that in 469 African-American patients with type 1 diabetes a high dietary sodium intake was associated with a higher 6 year incidence of macular oedema [12]. However, the authors used an FFQ to estimate dietary salt intake and potassium intake was not taken into account. High potassium intake has been shown to exert an inhibitory effect on sodium sensitivity [13] and high sodium intake in combination with low potassium intake results in further increases in BP [13]. However, the association between potassium intake and microvascular complications in type 1 diabetes has not yet been established.

We investigated the associations between sodium and potassium intake, estimated from 24 h urine samples, and the prevalence of microalbuminuria, macroalbuminuria, and non-proliferative and proliferative retinopathy in a European cohort of individuals with type 1 diabetes.

## Methods

### Study population

We used data from the EURODIAB Prospective Complications Study, a European based prospective cohort study that has been described in detail elsewhere [14, 15]. In brief, baseline investigations were performed between 1989 and 1991 on 3,250 patients with type 1 diabetes, defined as clinical diagnosis made before the age of 36 years, and needing continuous insulin therapy within 1 year of diagnosis. Patients aged 15–60 years were recruited from 31 centres in 16 European countries. Sample selection was stratified by sex, age group and duration of diabetes to ensure sufficient representation in all categories. These patients were invited for a follow-up examination on average 7–9 years after the baseline examinations. Of the 3,250 included patients, 1,880 (57.8%) returned for re-examination. We performed cross-sectional analyses on these follow-up data (as urine samples of the baseline investigations were no longer available).

Ethics committee approval conforming to the Declaration of Helsinki was obtained at each centre and all participants provided written informed consent.

### Urinary sodium and potassium excretion

We measured urinary sodium and potassium concentrations in one (*n* = 119) or two (*n* = 1,093) 24 h urine samples of 1,212 individuals from the EURODIAB follow-up measurement in 1997 by means of an ion-selective electrode (Cobas 6000; Roche Diagnostics, Tokyo, Japan). We multiplied 24 h urinary sodium excretion (in g/day) by 2.5 to estimate the dietary salt intake (in g/day).

### Microvascular complications

The AER was measured from duplicate 24 h urine collections using an immunoturbidimetric method that included goat anti-human albumin antisera (Sanofi Diagnostics Pasteur, Chaska, MN, USA) and human serum albumin standards (ORHA 20/21 grade HSA; Behring Diagnostics, Hoechst UK, Hounslow, UK) [16]. Microalbuminuria and macroalbuminuria were defined as AER 20–200 μg/min and >200 μg/min, respectively. The GFR was estimated using the Chronic Kidney Disease Epidemiology Collaboration (CKD-EPI) equation [17].

Retinopathy was centrally assessed from retinal photographs by a trained reader of colour retinal photographs using a system of 45° field grading standards for the assessment of retinopathy that was developed for the EURODIAB Prospective Complications Study. Non-proliferative retinopathy was defined as the presence of one or more microaneurysms, haemorrhages and/or hard exudates. Proliferative retinopathy was defined as any new vessel, fibrous proliferations, pre-retinal haemorrhage, vitreous haemorrhage or photocoagulation scar [18].

### Covariables

Weight and height were measured with indoor clothing without shoes and BMI was calculated as weight divided by height squared. Smoking habits were ascertained by questionnaire and individuals were categorised into never smokers, ex-smokers and current smokers. Self-reported physical activity was assessed by a questionnaire. The weekly metabolic equivalent (MET-h) corresponds to the weekly amount of time spent in each sporting activity multiplied by the corresponding MET value. Physical activity was then categorised into none, low (less than or equal to the sex-specific median; 9.6 MET-h/week for men and 7.3 MET-h/week for women) and high (greater than the sex-specific median).

A standardised 3 day dietary record was employed for dietary assessment, as previously described [19]. Briefly, local dietitians instructed individuals on how to record all food and beverages consumed over 3 days (two workdays and a Sunday) with the help of standardised food portion sizes. The local dietitian reviewed each completed record with each individual. Then, with the use of a centrally prepared EURODIAB food list, the records were analysed to quantify the amounts of total energy, total protein (animal and vegetable protein), total fat (saturated and unsaturated fat), total carbohydrate, fibre and alcohol intake. Alcohol consumption was categorised into none, low (less than or equal to the sex-specific median; 12.7 g/day for men and 7.1 g/day for women) and high (greater than the sex-specific median). We did not quantify dietary salt intake based on dietary records as this is considered unreliable due to recall bias, variations of sodium content of common food items, lack of information on sodium added during cooking or at the table and imprecision with estimating portion size [20, 21].

### Statistical analyses

All statistical analyses were carried out using the Predictive Analytics SoftWare, version 18.0 (SPSS IBM Corporation, Armonk, NY, USA), unless specified otherwise. Variables with a skewed distribution (i.e. GFR, triacylglycerols) were log_*e*_ transformed prior to all analyses.

General characteristics were compared between categories of urinary sodium excretion with the use of ANOVA or χ^2^ tests for continuous or categorical data, respectively.

In all, 378 individuals (31% of the total population) had missing values for one or more of the covariables. The percentage of missing values per variable varied from 0.1% (physical activity) to 17% (dietary intake). We used multiple imputation chained equations to impute those missing values rather than perform complete case analyses in order to decrease bias and increase the power of the analyses [22]. With this procedure, the imputation model of a single missing variable used the information from all the other variables considered as predictors by appropriate regression models (i.e. linear or logistic if variable to be imputed was continuous or dichotomous, respectively). We generated five imputed datasets, which were used to fit the regression models of interest. The results reported were those retrieved from pooled analyses on all five imputed datasets.

We used logistic regression analyses to investigate the associations between dietary salt intake and potassium excretion on the one hand and the prevalence of microvascular complications on the other, after adjustment first for age and sex (model 1), then additionally for BMI, smoking, urinary potassium (in the case of sodium), urinary sodium (in the case of potassium) and use of antihypertensive medication (model 2), and further for physical activity and total energy, protein, saturated fat, fibre and alcohol intake (model 3). We investigated these associations both with dietary salt intake divided into categories of 7.5–10 and >10 g/day vs <7.5 g/day, and with dietary salt intake as a continuous variable. We added quadratic terms of salt intake to our models to statistically examine potential non-linearity. To increase the power of our analyses, we present the analyses with salt intake as a continuous variable whenever no consistent non-linear associations between dietary salt intake and microvascular complications were found.

All regression analyses were conducted in the whole study population and also in the subgroup of individuals without prevalent CVD and/or antihypertensive medication, because individuals with prior CVD and/or known (treated) hypertension may have received dietary advice to reduce their salt intake, which may mask any associations. We additionally tested for interaction by variables that may modify dietary salt sensitivity at a given intake, i.e. urinary potassium excretion, kidney function and BMI, on the multiplicative scale by adding product terms between these variables and urinary sodium excretion to the fully adjusted models and on the additive scale by calculating the relative excess risk due to interaction (RERI) [23]. CIs of the RERI were estimated by using a bootstrap method with 10,000 bootstrap samples in R statistical software, version 2.15.2 (http://www.r-project.org) [23]. We stratified our analyses whenever significant interactions were found.

We performed sensitivity analyses to investigate the robustness of significant associations between dietary salt intake and microvascular complications by excluding individuals with potential under or over urine collections. Under or over collections of 24 h urine samples were defined as the lower and upper 2.5% of the difference between the measured GFR ([urine creatinine]*24 h urine volume/[serum creatinine]) and estimated GFR (using the CKD-EPI equation). We performed these sensitivity analyses in a subgroup in which urine creatinine concentrations were available (*n* = 484) [24].

## Results

The correlation between the two 24 h urine samples was *r* = 0.60 (*p* < 0.001) for sodium excretion and *r* = 0.56 (*p* < 0.001) for potassium excretion, and the correlation between urinary sodium and potassium excretion was *r* = 0.44 (*p* < 0.001). The average 24 h urinary sodium excretion rate was 4.0 g/day, which roughly corresponds to a dietary salt intake of 9.9 g/day.

Table 1 shows the general characteristics of the 1,212 individuals of the EURODIAB study population at follow-up (1997) with available data on urinary sodium and potassium excretion. Data are presented for the total population (*n* = 1,212) and stratified according to categories of dietary salt intake.

**Table 1:** General characteristics of the EURODIAB study

	Total population (*n* = 1,212)	Dietary salt intake			
		<7.5 g/day (*n* = 352)	7.5–10 g/day (*n* = 347)	>10 g/day (*n* = 513)	*p*_trend_
Age (years)	39.9 ± 9.8	39.6 ± 9.4	39.5 ± 9.6	40.4 ± 10.2	0.21
Male sex (%)	51	43	45	61	<0.001
BMI (kg/m^2^)	24.7 ± 3.3	24.0 ± 2.9	24.7 ± 3.5	25.1 ± 3.3	<0.001
HbA_1c_ (%) (mmol/mol)	8.4 ± 1.5 (68 ± 16)	8.6 ± 1.5 (70 ± 16)	8.3 ± 1.4 (67 ± 15)	8.4 ± 1.5 (68 ± 16)	0.31
Duration of diabetes (years)	21.8 ± 9.3	21.9 ± 9.0	21.7 ± 9.7	21.7 ± 9.2	0.78
Total cholesterol (mmol/l)	5.3 ± 1.1	5.3 ± 1.1	5.2 ± 1.0	5.4 ± 1.2	0.37
LDL cholesterol (mmol/l)	3.2 ± 1.0	3.2 ± 0.9	3.0 ± 0.9	3.3 ± 1.1	0.23
HDL cholesterol (mmol/l)	1.6 ± 0.4	1.6 ± 0.4	1.6 ± 0.5	1.6 ± 0.4	0.21
Triacylglycerols (mmol/l)	1.0 (0.8–1.4)	1.0 (0.8–1.4)	0.9 (0.8–1.3)	1.0 (0.8–1.4)	0.51
Smoking (never/ex/current) (%)	42/29/29	43/29/28	46/27/27	39/31/30	0.14
Systolic BP (mmHg)	121 ± 19	120 ± 19	122 ± 19	122 ± 19	0.25
Diastolic BP (mmHg)	74 ± 12	73 ± 12	74 ± 12	75 ± 12	0.059
Use of antihypertensive medication (%)	25	27	22	25	0.57
Use of ACE inhibitors (%)	21	22	17	22	0.94
Use of diuretics (%)	4	5	3	4	0.20
Estimated GFR (ml/min)	102 (90–112)	102 (86–111)	103 (90–111)	103 (92–113)	0.049
Urinary volume (l)	1.8 ± 0.7	1.6 ± 0.6	1.7 ± 0.6	2.1 ± 0.7	<0.001
Urinary creatinine (mmol)^a^	11.7 ± 3.9	10.0 ± 3.4	11.3 ± 3.6	13.3 ± 3.8	<0.001
CVD (%)	13	13	12	13	0.66
Albuminuria (normo/micro/macro) (%)	71/17/12	73/14/13	76/15/9	67/20/13	0.11
Retinopathy (none/non-proliferative/proliferative) (%)	31/48/21	31/47/22	33/44/23	29/52/19	0.75
Urinary sodium excretion (g/day)	3.96 ± 1.60	2.29 ± 0.53	3.49 ± 0.28	5.43 ± 1.25	NA
Urinary sodium excretion (mmol/day)	172 ± 69	100 ± 23	152 ± 12	236 ± 54	NA
Urinary potassium excretion (g/day)	2.91 ± 1.09	2.40 ± 0.90	2.80 ± 0.95	3.34 ± 1.14	<0.001
Urinary potassium excretion (mmol/day)	75 ± 28	62 ± 23	72 ± 24	86 ± 29	<0.001
Physical activity (0/≤median^b^/ >median) (%)	60/20/19	61/22/17	57/24/19	62/16/21	0.70
Total energy intake (kJ/day)	9856 ± 2887	9147 ± 2620	9590 ± 2718	10522 ± 3030	<0.001
Protein intake (g/day)	97.8 ± 29.1	90.5 ± 27.1	93.8 ± 26.7	105.6 ± 30.2	<0.001
Alcohol intake (0/≤median^c^, >median [%])	45/27/28	42/27/31	49/25/26	44/29/27	0.55
Saturated fat intake (g/day)	35.8 ± 15.3	33.2 ± 14.4	35.1 ± 14.2	37.9 ± 16.2	<0.001
Fibre intake (g/day)	19.9 ± 8.5	18.1 ± 7.2	19.2 ± 8.0	21.5 ± 9.4	<0.001

Data are presented as means ± SD, medians (interquartile range) or percentages, as appropriate

^a^urinary creatinine was only available in *n* = 489

^b^sex-specific medians for physical activity were 9.6 MET-h/week for men and 7.3 MET-h/week for women

^c^sex-specific medians for alcohol intake were 12.7 g/day for men and 7.1 g/day for women

Individuals with higher salt intake were more often men and had on average higher values of BMI, urinary volume, urinary creatinine and potassium excretion, and higher total energy and protein intake (Table 1).

After adjustment for age and sex, we found no associations between dietary salt intake (per 1 g/day) and systolic BP (–0.14 mmHg [95% CI –0.39, 0.12]) or diastolic BP (0.02 [–95% CI 0.15, 0.19]).

### Associations between dietary salt intake and microvascular complications

After adjustment for age and sex, >10 g/day salt intake, when compared with <7.5 g/day salt intake, was positively associated with the prevalence of microalbuminuria (OR 1.46 [95% CI 1.00, 2.13]). After full adjustment a similar association with microalbuminuria was found (OR 1.40 [95% CI 0.92, 2.15]), although this was no longer significant (ESM Table 1). When individuals with CVD and/or those who used antihypertensive medication were excluded we found a similar positive, although non-significant, association between >10 g/day salt intake and microalbuminuria (OR 1.26 [95% CI 0.74, 2.15]) and macroalbuminuria (OR 1.34 [95% CI 0.55, 3.27]) compared with <7.5 g/day salt intake (ESM Table 2). We found no consistent associations between 7.5–10 g/day or >10 g/day salt intake and non-proliferative or proliferative retinopathy, when compared with <7.5 g/day salt intake either in the total population (ESM Table 1) or after exclusion of individuals with CVD and/or antihypertensive medication (ESM Table 2). In addition, we found no significant associations between the quadratic terms of salt intake and microvascular complications (all *p* > 0.46). Taken together, these analyses provide no consistent evidence for non-linear associations between salt intake and microvascular complications. We therefore present all further analyses per 1 g/day higher salt intake.

After adjustment for age and sex, 1 g/day higher dietary salt intake was positively associated with microalbuminuria (OR 1.06 [95% CI 1.02, 1.10]) but not with macroalbuminuria (OR 1.00 [95% CI 0.95, 1.04]), non-proliferative retinopathy (OR 1.02 [95% CI 0.98, 1.05]) or proliferative retinopathy (OR 0.99 [95% CI 0.94, 1.03]) (Table 2). After excluding individuals with CVD and/or antihypertensive medication (*n* = 418), we found a similar positive association with microalbuminuria (OR 1.05 [95% CI 1.00, 1.11]) and also, although not significantly so, with macroalbuminuria (OR 1.04 [95% CI [0.96; 1.13]), with no associations for non-proliferative retinopathy (OR 1.01 [95% CI 0.97, 1.05]) or proliferative retinopathy (OR 1.02 [95% CI 0.95, 1.09]) (Table 3). Essentially the same results were found in the fully adjusted models (Tables 2 and 3).

**Table 2:** Continuous associations between dietary salt intake and prevalent albuminuria and retinopathy

	Microalbuminuria (205/1,068)			Macroalbuminuria (143/1,006)			Non-proliferative retinopathy (507/833)			Proliferative retinopathy (224/550)		
Model	OR	95% CI	*p*	OR	95% CI	*p*	OR	95% CI	*p*	OR	95% CI	*p*
1	1.06	1.02, 1.10	0.003	1.00	0.95, 1.04	0.88	1.02	0.98, 1.05	0.34	0.99	0.94, 1.03	0.53
2	1.06	1.01, 1.10	0.011	1.00	0.94, 1.06	0.95	1.00	0.96, 1.04	0.94	1.02	0.96, 1.08	0.64
3	1.06	1.01, 1.10	0.013	0.99	0.94, 1.05	0.82	1.00	0.96, 1.04	0.84	1.02	0.95, 1.08	0.65

OR indicates the odds of prevalent albuminuria or retinopathy per g/day salt intake

Model 1: adjusted for age and sex

Model 2: model 1 + BMI, smoking (never/ex/current), urinary potassium excretion and use of antihypertensive medication

Model 3: model 2 + physical activity (0/≤sex-specific median/>sex-specific median), total energy intake, protein intake, saturated fat intake, fibre intake, alcohol intake (0/≤sex-specific median/>sex-specific median)

**Table 3:** Continuous associations between dietary salt intake and prevalent albuminuria and retinopathy in individuals without CVD who did not use antihypertensive medication

	Microalbuminuria (116/794)			Macroalbuminuria (41/719)			Non-proliferative retinopathy (367/642)			Proliferative retinopathy (92/367)		
Model	OR	95% CI	*p*	OR	95% CI	*p*	OR	95% CI	*p*	OR	95% CI	*p*
1	1.05	1.00, 1.11	0.049	1.04	0.96, 1.13	0.36	1.01	0.97, 1.05	0.66	1.02	0.95, 1.09	0.63
2	1.04	0.98, 1.10	0.17	1.05	0.96, 1.15	0.30	0.98	0.94, 1.03	0.44	1.02	0.95, 1.10	0.54
3	1.04	0.99, 1.10	0.14	1.05	0.96, 1.16	0.30	0.98	0.93, 1.02	0.32	1.02	0.95, 1.10	0.59

OR indicates the odds of prevalent albuminuria or retinopathy per g/day salt intake

Model 1: adjusted for age and sex

Model 2: model 1 + BMI, smoking (never/ex/current) and urinary potassium excretion

Model 3: model 2 + physical activity (0/≤sex-specific median/>sex-specific median), total energy intake, protein intake, saturated fat intake, fibre intake, alcohol intake (0/≤sex-specific median/>sex-specific median)

We found no consistent significant additive or multiplicative interactions between salt intake and urinary potassium excretion or kidney function in the associations with microvascular complications (data not shown). However, in the association between salt intake and microalbuminuria we did find an interaction with BMI (*p* for multiplicative interaction 0.050 and RERI 0.02 [95% CI –0.02, 0.07)] such that the association was stronger in individuals with BMI >25 kg/m^2^ (OR 1.11 [95% CI 1.04, 1.18]) than in those with BMI <25 kg/m^2^ (OR 1.03 [95% CI 0.97, 1.09]).

### Associations between urinary potassium excretion and microvascular complications

After adjustment for age and sex we found no significant associations between urinary potassium excretion (in g/day) and microalbuminuria (OR 1.09 [95% CI 0.95, 1.25]), macroalbuminuria (OR 0.98 [95% CI 0.83, 1.16]) and non-proliferative (OR 1.12 [95% CI 0.98, 1.27]) or proliferative (OR 0.87 [95% CI 0.73, 1.04]) retinopathy (Table 4). Similarly, no significant associations were found after full adjustment (Table 4) or when individuals with CVD and/or those who used antihypertensive medication were excluded (Table 5). In addition, we found no consistent significant additive or multiplicative interactions of urinary potassium excretion with urinary sodium excretion, kidney function or BMI (data not shown).

**Table 4:** Continuous associations between urinary potassium excretion and prevalent albuminuria and retinopathy

	Microalbuminuria (205/1,068)			Macroalbuminuria (143/1,006)			Non-proliferative retinopathy (507/833)			Proliferative retinopathy (224/550)		
Model	OR	95% CI	*p*	OR	95% CI	*p*	OR	95% CI	*p*	OR	95% CI	*p*
1	1.09	0.95, 1.25	0.20	0.98	0.83, 1.16	0.79	1.12	0.98, 1.27	0.10	0.87	0.73, 1.04	0.13
2	1.03	0.88, 1.20	0.76	1.04	0.85, 1.28	0.71	1.10	0.94, 1.28	0.24	0.84	0.67, 1.05	0.12
3	1.03	0.88, 1.22	0.68	1.07	0.86, 1.32	0.57	1.11	0.95, 1.30	0.20	0.88	0.69, 1.12	0.29

OR indicates the odds of prevalent albuminuria or retinopathy per g/day potassium excretion

Model 1: adjusted for age and sex

Model 2: model 1 + BMI, smoking (never/ex/current), urinary sodium excretion and use of antihypertensive medication

Model 3: model 2 + physical activity (0/≤sex-specific median/>sex-specific median), total energy intake, protein intake, saturated fat intake, fibre intake, alcohol intake (0/≤sex-specific median/>sex-specific median)

**Table 5:** Continuous associations between urinary potassium excretion and prevalent albuminuria and retinopathy in individuals without CVD who did not use antihypertensive medication

	Microalbuminuria (114/779)			Macroalbuminuria (40/705)			Non-proliferative retinopathy (360/633)			Proliferative retinopathy (92/365)		
Model	OR	95% CI	*p*	OR	95% CI	*p*	OR	95% CI	*p*	OR	95% CI	*p*
1	1.12	0.95, 1.33	0.18	0.92	0.68, 1.25	0.60	1.15	0.99, 1.33	0.074	0.88	0.69, 1.14	0.34
2	1.06	0.88, 1.28	0.55	0.85	0.60, 1.20	0.35	1.15	0.97, 1.36	0.10	0.84	0.63, 1.11	0.21
3	1.07	0.88, 1.30	0.52	0.82	0.57, 1.17	0.28	1.16	0.98, 1.39	0.090	0.91	0.68, 1.22	0.52

OR indicates the odds of prevalent albuminuria or retinopathy per g/day potassium excretion

Model 1: adjusted for age and sex

Model 2: model 1 + BMI, smoking (never/ex/current) and urinary sodium excretion

Model 3: model 2 + physical activity (0/≤sex-specific median/>sex-specific median), total energy intake, protein intake, saturated fat intake, fibre intake, alcohol intake (0/≤sex-specific median/>sex-specific median)

### Additional analyses

Use of salt intake <5.0 g/day (*n* = 85) instead of <7.5 g/day (*n* = 352) as a reference category did not materially change our results (data not shown).

### Sensitivity analyses

In the subgroup of 484 individuals who had urine creatinine values available we found a positive association between dietary salt intake and microalbuminuria (OR 1.12 [95% CI 1.04, 1.22] after full adjustment), which was similar after exclusion of individuals with potential under or over 24 h urine collections (*n* = 24; OR 1.12 [95% CI 1.03, 1.22]).

Complete case analyses showed similar results to the multiple imputation analyses as presented throughout the manuscript.

## Discussion

The present study showed that in individuals with type 1 diabetes, higher dietary salt intake, as estimated by 24 h urinary sodium excretion, was associated with a higher prevalence of microalbuminuria, an association that was more pronounced in overweight individuals, but not with macroalbuminuria or retinopathy. In addition, no significant associations were found between urinary potassium excretion and the prevalence of microvascular complications.

Previous studies on the cross-sectional association between dietary salt intake and microalbuminuria in the general population have been conflicting, that is both no [4, 8] and positive [5–7, 9] associations have been described. Interestingly, these positive associations were more pronounced in individuals with a higher BMI [5] or were found only in obese individuals [7]. The present results of a more pronounced positive association between dietary salt intake and microalbuminuria in individuals with type 1 diabetes and a BMI >25 kg/m^2^ support previous findings [5, 6]. Overweight and obesity have been associated with both salt sensitivity [7, 25] and increased glomerular filtration fraction and GFR [26], which might explain the stronger associations between salt intake and microalbuminuria.

In contrast, two studies in individuals with type 2 [10] or type 1 diabetes [11] found inverse (or J-shaped) associations between dietary salt intake and prevalent microalbuminuria [10] or incident end-stage renal disease [11]. These findings are in line with several other recent studies that showed J-shaped associations between dietary salt intake and incident CVD and mortality in general [27], as well as in high-risk populations [28] and in individuals with type 1 [11] and type 2 diabetes [29]. However, several methodological aspects of these studies have been criticised, such as the use of a single 24 h urine sample, which may poorly represent usual sodium intake at the individual level [30, 31], potential problematic completeness of urine collection [30] and unmeasured and residual confounding [32]. Furthermore, the low salt-intake groups may have included persons who followed a low salt diet, which may have been recommended to those with hypertension or otherwise increased CVD risk, which could have biased the findings [31]. In addition, in the present study we found no evidence for such non-linear associations between dietary salt intake and microvascular complications. Whether or not low dietary salt intake is associated with increased cardiovascular risk and/or microvascular complications thus remains to be established.

Potassium has been shown to exert an inhibitory effect on sodium sensitivity [13], and the association between sodium intake and microvascular complications may thus be modified by potassium intake. In addition, high sodium intake in combination with low potassium intake results in further excess of sodium in the body and higher BP [13]. However, we did not find evidence to support an effect-modifying role of potassium excretion in the association between sodium excretion and microvascular complications nor that of an (inverse) association between potassium excretion and microvascular complications in individuals with type 1 diabetes. In addition, although individuals with decreased kidney function are more commonly BP salt-sensitive, and kidney function may thus modify the association between dietary salt intake and microvascular complications, we found no evidence to support this contention. However, we may have lacked power to show this association, and larger and prospective studies are needed to fully address these issues.

Increasing dietary salt intake increases BP in salt-sensitive individuals both with [33] and without [34] type 1 diabetes. In individuals with type 1 diabetes, elevated BP has been associated with the development of microalbuminuria [35], which may result from an increase in the ratio of GFR to the renal plasma flow, i.e. an increased glomerular filtration fraction, with a resultant increase in proteinuria [34]. In the current study, however, higher dietary salt intake was not associated with elevated BP. Individuals with elevated BP may have changed their dietary habits to reduce salt intake, which may have masked these cross-sectional associations. However, the association between dietary salt intake and microalbuminuria may also be mediated by processes other than increases in BP. High salt intake may activate the local renin–angiotensin–aldosterone system in the vessels as well as in the kidney [36], stimulating the secretion of aldosterone, and this may have detrimental effects on endothelium-dependent dilation [37] as it may stimulate central sympathetic outflow creating a hyperadrenergic state [38] and the formation of reactive oxygen species suppressing nitric oxide availability [39]. Similar effects of aldosterone may be exerted via increased signalling of the mineralocorticoid receptor resulting from or in combination with high salt intake [40]. However, we cannot draw any conclusions of a cause-and-effect relationship between salt intake and microalbuminuria based on the current cross-sectional study.

Although the current study is the largest study investigating the association between dietary salt intake and (early) microvascular complications in individuals with type 1 diabetes so far, a limitation that needs to be addressed is the cross-sectional design. We cannot exclude that individuals with prior CVD and/or known (treated) hypertension have received dietary advice to reduce their salt intake, which may have masked the associations. We have therefore performed all analyses not only in the total population but also in the individuals without prevalent CVD and/or antihypertensive medication. However, the analyses in this subpopulation might have been underpowered due to low numbers of microvascular complication cases in particular of macroalbuminuria. Further investigation in larger, prospective studies is warranted to address the role of dietary salt intake in the development of microvascular complications in type 1 diabetes.

In conclusion, in individuals with type 1 diabetes, high dietary salt intake may be a risk factor for microalbuminuria, particularly in overweight individuals, and nutritional advice regarding salt intake may thus be important in preventing or slowing the rate of development of microvascular disease in individuals with type 1 diabetes. Further investigation in prospective studies is warranted to fully address the role of dietary salt intake in the development of microvascular complications in type 1 diabetes.

## Electronic supplementary material

Below is the link to the electronic supplementary material.

ESM Table 1 (PDF 264 kb)

ESM Table 2 (PDF 264 kb)

ESM list of study group members (PDF 262 kb)

## Abbreviations

CKD-EPI: Chronic Kidney Disease Epidemiology Collaboration
CVD: Cardiovascular disease
FFQ: Food frequency questionnaire
MET-h: Weekly metabolic equivalent
RERI: Relative excess risk due to interaction
