# Supplementary material for: Higher dietary salt intake is associated with microalbuminuria, but not with retinopathy in individuals with type 1 diabetes: the EURODIAB Prospective Complications Study
Source: Diabetologia. 2014 Aug 30;57(11):2315–23. doi: 10.1007/s00125-014-3367-9 (PMC4181505; doi:10.1007/s00125-014-3367-9)
Supplement: Supplementary file 1 — (PDF 264 kb) [file 125_2014_3367_MOESM1_ESM.pdf]

ESM table 1 Associations between 'medium' and 'high' dietary salt intake (vs. 'low' salt intake) and prevalent albuminuria and retinopathy

		Microalbuminuria (205/1,068)			Macroalbuminuria (143/1,006)			Non-proliferative retinopathy (507/833)			Proliferative retinopathy (224/550)		
model	salt intake	OR	95% CI	*p*	OR	95% CI	*p*	OR	95% CI	*p*	OR	95% CI	*p*
1	7.5-10 g/day	0.99	0.65; 1.52	0.97	0.71	0.44; 1.16	0.18	0.91	0.63; 1.31	0.60	1.01	0.65; 1.58	0.96
	>10 g/day	1.46	1.00; 2.13	0.051	0.97	0.63; 1.48	0.87	1.12	0.79; 1.57	0.52	0.86	0.56; 1.31	0.47
2	7.5-10 g/day	1.01	0.65; 1.57	0.97	0.80	0.46; 1.38	0.42	0.85	0.58; 1.24	0.39	1.12	0.86; 1.47	0.66
	>10 g/day	1.42	0.93; 2.15	0.10	1.02	0.61; 1.70	0.95	0.96	0.66; 1.40	0.84	1.09	0.64; 1.84	0.75
3	7.5-10 g/day	1.00	0.64; 1.56	0.99	0.82	0.47; 1.45	0.50	0.81	0.55; 1.20	0.30	1.13	0.67; 1.93	0.64
	>10 g/day	1.40	0.92; 2.15	0.12	0.99	0.57; 1.70	0.96	0.92	0.63; 1.36	0.69	1.05	0.60; 1.82	0.87

OR, odds ratio indicates the odds of prevalent albuminuria or retinopathy for salt intake of 7.5-10 and >10 g/day vs. salt intake of <7.5 g/day. Model 1: adjusted for age and sex; Model 2: model 1 + BMI, smoking (never, ex, current), urinary potassium excretion and use of antihypertensive medication; Model 3: model 2 + physical activity (0, ≤sex-specific median, >sex-specific median), total energy intake, protein intake, saturated fat intake, fibre intake, alcohol intake (0, ≤sex-specific median, >sex-specific median).
