# Supplementary material for: Higher dietary salt intake is associated with microalbuminuria, but not with retinopathy in individuals with type 1 diabetes: the EURODIAB Prospective Complications Study
Source: Diabetologia. 2014 Aug 30;57(11):2315–23. doi: 10.1007/s00125-014-3367-9 (PMC4181505; doi:10.1007/s00125-014-3367-9)
Supplement: Supplementary file 2 — (PDF 264 kb) [file 125_2014_3367_MOESM2_ESM.pdf]

ESM table 2 Associations between 'medium' and 'high' dietary salt intake (vs. 'low' salt intake) and prevalent albuminuria and retinopathy in individuals without CVD and who do not use antihypertensive medication

		Microalbuminuria (116/794)			Macroalbuminuria (41/719)			Non-proliferative retinopathy (367/642)			Proliferative retinopathy (92/367)		
model	salt intake	OR	95% CI	*p*	OR	95% CI	*p*	OR	95% CI	*p*	OR	95% CI	*p*
1	7.5-10 g/day	0.96	0.56; 1.65	0.87	0.98[a]	0.41; 2.37	0.97	1.01	0.67; 1.52	0.97	1.37	0.73; 2.55	0.33
	>10 g/day	1.33	0.81; 2.18	0.26	1.25[b]	0.56; 2.77	0.59	0.99	0.68; 1.46	0.97	1.07	0.58; 1.98	0.83
2	7.5-10 g/day	0.92	0.53; 1.59	0.76	1.09[a]	0.45; 2.68	0.85	0.94	0.62; 1.43	0.77	1.38	0.74; 2.60	0.31
	>10 g/day	1.19	0.71; 2.02	0.51	1.35[b]	0.58; 3.17	0.49	0.82	0.54; 1.24	0.34	1.09	0.57; 2.09	0.80
3	7.5-10 g/day	0.94	0.54; 1.64	0.83	1.19[a]	0.47; 2.98	0.71	0.90	0.59; 1.39	0.64	1.42	0.73; 2.75	0.30
	>10 g/day	1.26	0.74; 2.15	0.40	1.34[b]	0.55; 3.27	0.52	0.77	0.50; 1.19	0.24	0.98	0.49; 1.97	0.95

OR, odds ratio indicates the odds of prevalent albuminuria or retinopathy for salt intake of 7.5-10 and >10 g/day vs. salt intake of <7.5 g/day. Model 1: adjusted for age and sex; Model 2: model 1 + BMI, smoking (never, ex, current), urinary potassium excretion and use of antihypertensive medication; Model 3: model 2 + physical activity (0, ≤sex-specific median, >sex-specific median), total energy intake, protein intake, saturated fat intake, fibre intake, alcohol intake (0, ≤sex-specific median, >sex-specific median).

[a]n=11 vs. 10 cases

[b]n=20 vs. 10 cases
