# Supplementary material for: Higher dietary salt intake is associated with microalbuminuria, but not with retinopathy in individuals with type 1 diabetes: the EURODIAB Prospective Complications Study
Source: Diabetologia. 2014 Aug 30;57(11):2315–23. doi: 10.1007/s00125-014-3367-9 (PMC4181505; doi:10.1007/s00125-014-3367-9)
Supplement: Supplementary file 3 — (PDF 262 kb) [file 125_2014_3367_MOESM3_ESM.pdf]

ESM list of study group members

The EURODIAB Prospective Complications Study Group

B. Karamanos, A. Kofinis, K. Petrou, Hippokration Hospital, Athens, Greece; F. Giorgino, G. Picca, A. Angarano, G. de Pergola, L. Laviola, R. Giorgino, Internal Medicine, Endocrinology and Metabolic Diseases, Department of Emergency and Organ Transplantation, University of Bari, Bari, Italy; C. Ionescu-Tirgoviste, A. Coszma, C. Guja, Clinic of Diabetes, Nutrition and Metabolic Diseases, Bucharest, Romania; M. Songini, A. Casu, M. Pedron, S. Pintus, M. Fossarello, Diabetes Unit Ospedale San Michele, Cagliari, Italy; J. B. Ferriss, G. Grealy, D. O'Keefe, Cork University Hospital, Cork, Ireland; M. Toeller, C. Arden, Diabetes Research Institute, Heinrich-Heine University, Duesseldorf, Germany; R. Rottiers, C. Tuyttens, H. Priem, University Hospital of Gent, Belgium; P. Ebeling, M. Kylliäinen , V. A. Koivisto, University Hospital of Helsinki, Finland; B. Idzior-Walus, J. Sieradzki, K. Cyganek, B. Solnica, Department of Metabolic Diseases, Jagiellonian University, Krakow, Poland; H. H. P. J. Lemkes, J. C. Lemkes-Stuffken, Leiden University Medical Centre, the Netherlands; J. Nunes-Correa, M. C. Rogado, L. Gardete-Correia, M. C. Cardoso, A. Silva, J. Boavida, M. Machado Sa Marques, Portuguese Diabetic Association, Lisbon, Portugal; G. Michel, R. Wirion, S. Cardillo, Centre Hospitalier, Luxembourg; G. Pozza, R. Mangili, V. Asnaghi, Ospedale San Raffaele, Milan, Italy; E. Standl, B. Schaffler, H. Brand, A. Harms, City Hospital Schwabing, Munich, Germany; D. Ben Soussan, O. Verier-Mine, P. Fallas, M. C. Fallas , Centre Hospitalier de Valenciennes, France; J. H. Fuller, J. Holloway, L. Asbury, D. J. Betteridge, University College London, UK; G. Cathelineau, A. Bouallouche, B. Villatte Cathelineau, Hospital Saint-Louis, Paris, France; F. Santeusanio, G. Rosi, V. D'Alessandro, C. Cagini, P. Bottini, G. P. Reboldi, Dipartimento di Medicina Interna, Perugia, Italy; R. Navalesi, G. Penno, S. Bandinelli, R. Miccoli, M. Nannipieri, Dipartimento di Endocrinologia e Metabolismo, Pisa, Italy; G. Ghirlanda, C. Saponara, P. Cotroneo, A. Manto, A. Minnella, Universita Cattolica del Sacro Cuore, Rome, Italy; J. D. Ward, S. Tesfaye, S. Eaton, C. Mody, Royal Hallamshire Hospital, Sheffield, UK; M. Borra, P. Cavallo Perin, S. Giunti, G. Grassi, G. F. Pagano, M. Porta, R. Sivieri, F. Vitelli, M. Veglio, Dipartimento di Medicina Interna, Università di Torino and ASO TO/CRF/Maria Adelaide, Turin, Italy; N. Papazoglou, G. Manes, General Hospital Papageorgiou, Diabetes Unit, Thessaloniki, Greece; M. Muggeo, M. Iagulli, V. Cacciatori, V. Cattedra di Malattie del Metabolismo, Verona, Italy; K. Irsigler, H. Abrahamian, Hospital Vienna Lainz, Austria; S. Walford, J. Sinclair, S. Hughes, V. McLelland, J. Ward, New Cross Hospital, Wolverhampton, UK; G. Roglic, Z. Metelko, Z. R. Pepeonik, Vuk Vrhovac Institute for Diabetes, Zagreb, Croatia

Steering committee members

J. H. Fuller (London), B. Karamanos, Chairman (Athens), A.-K. Sjolie (Odense), N. Chaturvedi (London), M. Toeller (Duesseldorf), G. Pozza Co-chairman (Milan), B. Ferriss (Cork), M. Porta (Turin), R. Rottiers (Gent), G. Michel (Luxembourg)

Co-ordinating centre

J. H. Fuller, N. Chaturvedi, J. Holloway, D. Webb, L. Asbury, University College London, UK

Central laboratories

G.-C. Viberti, R. Swaminathan, P. Lumb, A. Collins, S. Sankaralingham, M.A. Crook, Guy's and St Thomas Hospital, London, UK

Retinopathy Grading Centre

S. Aldington, T. Mortemore, H. Lipinski, Royal Postgraduate Medical School of Imperial College London, London, UK

Nutrition Co-ordinating Centre

M. Toeller, W.A. Scherbaum, F.A. Gries, Heinrich-Heine-University, Diabetes Research Institute and Department of Endocrinology, Diabetology and Rheumatology, Duesseldorf, Germany
